# Psychological stress, cognitive decline and the development of dementia in amnestic mild cognitive impairment

**DOI:** 10.1038/s41598-020-60607-0

**Published:** 2020-02-27

**Authors:** Rebecca Sussams, Wolff Schlotz, Zoe Clough, Jay Amin, Sharon Simpson, Amelia Abbott, Rebecca Beardmore, Richard Sharples, Rachel Raybould, Keeley Brookes, Kevin Morgan, David Culliford, Clive Holmes

**Affiliations:** 1 Faculty of Medicine, Clinical Experimental Sciences, University of Southampton, Southampton, UK; 2 Memory Assessment and Research Centre; Moorgreen Hospital; Southern Health Foundation Trust, Southampton, UK; 3 MaxPlanckInstitut for Empirical Aesthetics, Frankfurt am Main, Germany; 4 St Mary’s Community Health Campus, Solent health care trust, Portsmouth, UK; 5 Institute of Psychological Medicine and Clinical Neurosciences, Cardiff University, Cardiff, UK; 6 Human Genetics, School of Life Sciences, University of Nottingham, Nottingham, UK; 7 NIHR CLAHRC Wessex Data Science Hub, Faculty of Health Sciences, University of Southampton, Southampton, UK

**Keywords:** Predictive markers, Alzheimer's disease, Risk factors

## Abstract

To determine the relationship between psychological stress with cognitive outcomes in a multi-centre longitudinal study of people with amnestic mild cognitive impairment (aMCI) we assessed three parameters of psychological stress (Recent Life Changes Questionnaire (RLCQ); the Perceived Stress Scale (PSS) and salivary cortisol) and their relationship with rates of cognitive decline over an 18 month follow up period and conversion to dementia over a 5.5 year period. In 133 aMCI and 68 cognitively intact participants the PSS score was higher in the aMCI compared with control group but neither the RLCQ scores nor salivary cortisol measures were different between groups. In the aMCI group the RLCQ and the PSS showed no significant association with cognitive function at baseline, cognitive decline or with conversion rates to dementia but high salivary cortisol levels were associated with RLCQ scores and poorer cognitive function at baseline and lower rates of cognitive decline. No relationship was found between salivary cortisol levels and conversion rate to dementia. We conclude that psychological stress as measured by the RLCQ or PSS was not associated with adverse cognitive outcomes in an aMCI population and hypothesise that this may reflect diminished cortisol production to psychological stress as the disease progresses.

## Introduction

Longitudinal cohort studies have identified a number of risk factors for the progression of amnestic mild cognitive impairment (aMCI) towards the development of dementia. Age, gender^1^ and possession of the Apolipiprotein E (ApoE) ε4 allele^2^ are established demographic and genetic risk factors but psychosocial factors are also cited including low educational achievement^3^ and depressive illness^4^. High levels of psychological stress and other mental health problems are known to be higher in people with aMCI^5–7^. Psychological stress is an emerging risk factor for the development of aMCI towards dementia. Several longitudinal cohort studies have demonstrated a link between the experience of psychological stress and cognitive decline later in life^8–13^. Psychological stress as a risk factor for the development of aMCI or dementia is more contentious with some studies showing a positive relationship^14–17^ and others finding no relationship^18–20^.

Measures of stress differ from study to study with some studies utilising objective stress measures (e.g. adverse life events); some studies subjective stress measures (e.g. perceived stress or distress measures) and others biological measures (e.g. salivary cortisol) but no single study has examined all three measures. Psychosocial stress, measured by adverse life events, is emerging as a possible risk factor for the development of cognitive impairment in older persons^12,13,21,22^. Other studies have suggested that the symptoms of chronic stress (distress) increase the risk of developing aMCI^5,16,23^. The biological underpinning of chronic stress has focussed on cortisol production and its potential for hippocampal damage^24,25^. Cross sectional studies of the elderly show that high cortisol levels are associated with increased cognitive impairment^19,26^. To date, one small longitudinal study in a mixed Mild Cognitive Impairment population has suggested that adverse life events may be associated with increased cognitive decline but found no relationship between adverse life events and cortisol levels and unexpectedly found a protective effect of cortisol on cognitive decline^21^.

We hypothesised that our primary measure of stress, the Recent Life Changes Questionnaire (RLCQ)^27^; and secondary measures (the Perceived Stress Scale (PSS)^28^ and biological measures of stress (salivary cortisol measures)) would be associated with a faster rate of cognitive decline as measured by our primary outcome measure the Free and Cued Selective Reminding Test (FCSRT-IR)^29^. Secondary outcome measures included change in the Montreal Cognitive Assessment (MocA)^30^ and conversion rates to dementia.

## Results

### Baseline comparisons

All 201 participants completed baseline measures. Table 1 shows the demographics, baseline cognitive scores and ApoE ε4 carrier status of the participants by participant group. All participants were white, Caucasian. The aMCI group were older and less likely to be female than the control group. The frequency of the ApoE ε4 allele was higher in the aMCI group than the control group. The aMCI group scored lower than the control group on both the MOCA and FCSRT-IR cognitive test scores at baseline.

**Table 1:** Demographics and baseline cognitive measures by group.

Variable	Control (n = 68)	aMCI (n = 133)	Mean difference (95% CI) t-test or Χ² (d.f.)
Age (years (se))	68.4 (1.1)	77.6 (0.6)	−9.2 (−11.6 to −6.8), P <0.0001
Gender female (n (%))	47 (69%)	52 (39%)	Χ²(1) = 16.2, P <0.001
APOE- ε4 present (n (%)	17 (27%)	56 (46%)	Χ²(1) = 6.0, P = 0.014
FCSRT-IR (mean (s.e.)	47.6 (0.1)	39.0 (0.8)	8.6 (6.3 to 11.0), P <0.0001
MOCA (mean (s.e.)	28.0 (0.2)	22.9 (0.2)	5.1 (4.4 to 5.8), P <0.0001

ApoE ε4 = Apolipoprotein E ε4 allele carrier; MOCA = Montreal Cognitive Assessment; FCSRT-IR = Free and Cued Selective Reminding Test with immediate recall.

Table 2 shows the mean RLCQ, PSS scores and salivary cortisol levels at baseline by group. There was a significantly higher baseline PSS score in the MCI group compared with the control group which remained significant after correcting for age and gender differences; emphasising the high level of perceived stress in this group. The other baseline measures of stress did not show significant differences between groups after correction for age and gender. Although there was a non-significant (p = 0.05) trend for higher cortisol AUC levels in the aMCI group after correction for age and gender differences.

**Table 2:** Stress measures at baseline by group.

Variable	Control (n = 68)	aMCI (n = 133)	Mean difference (95% CI) t-test
RLCQ pts (se)	160.2 (16.2)	125.1 (9.1)	35.1 (1.2 to 69.1), P = 0.04 −7.9 (−47.4 to 31.6), P = 0.7
PSS pts (se)	12.1 (0.8)	14.6 (0.6)	2.5 (0.4 to 4.6), P = 0.02 3.4 (0.9 to 5.9), P = 0.001^c^
S1 (se) nmol/L	11.3 (1.1)	12.2 (0.6)	−0.9 (−3.1 to 1.2), P = 0.4 0.1 (−1.5 to 3.7), P = 0.4^c^
CAR (se) nmol/L	3.8 (1.0)	2.3 (0.6)	1.5 (−0.6 to 3.7,) P = 0.2 0.9 (−1.6 to 3.5,) P = 0.5^c^
Cortisol AUC (se) nmol/L	124.5 (7.5)	145.3 (8.8)	20.8 (−4.5 to 46.1), P = 0.1 29.2 (−0.3 to 58.7), P = 0.05^c^

RLCQ = Recent Life Changes Questionnaire; PSS = Perceived Stress Scale; S1 = Awakening cortisol sample; CAR = Cortisol awakening response; cortisol AUC = area under daytime cortisol curve. ^c^Corrected for age and gender.

**Figure 1:**
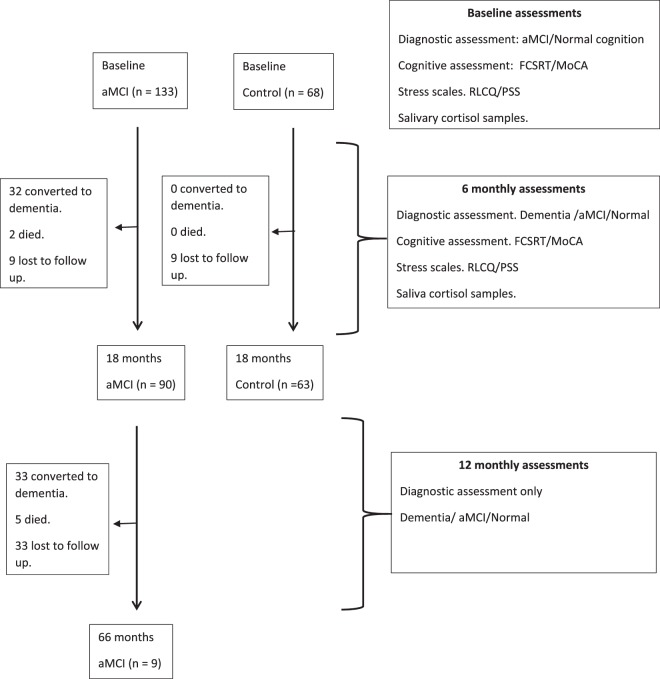
Strobe flow chart for participants in the ICOS study.

Figure 1 shows the flow of participants through the study. From the 68 control participants at baseline 66 participants completed the 6 month psychometric assessment; 66 participants completed the 12 month assessment and 63 participants completed the 18 month assessment. From the 133 aMCI participants at baseline 120 aMCI participants completed the 6 month psychometric assessment; 99 participants completed the 12 month assessment and 90 participants completed the 18 month assessment. Of a possible 4230 salivary samples 4133 (98% compliance) were collected during the 18 month assessment. 210 samples had an unreadable cortisol output; 204 samples were taken outside of the appropriate time range and 28 had cortisol levels that were extremely high (>100 nmol/L) leaving 3691 (87%) for final analysis. 78 of the 90 (87%) aMCI participants who had completed the 18 month study gave consent for annual diagnostic review. No salivary samples were taken after the first 18 months of the study.

### Relationship between psychometric stress measures and cortisol measures

The supplemental file shows the relationship between the first salivary cortisol measure taken as soon as possible after wakening (S1), the cortisol awakening response (CAR) measuring the post-awakening surge in cortisol occurring within 30 minutes after wakening and the area under the daytime cortisol curve (cortisol AUC), an estimate of the average daily cortisol exposure and the psychometric stress variables RLCQ and PSS and demographic variables. Higher average S1 cortisol, lower CAR and higher daily cortisol levels on the between-subject level (i.e. aggregated across visits) were associated with higher average PSS scores, but these associations were not found in models adjusted for age, gender, and group. No relationship was found with RLCQ scores on the between-subject level. On the within-subject level, higher individual S1 cortisol measures; lower CAR and higher daily cortisol measures were associated with higher individual RLCQ scores in the aMCI compared to the control group. This demonstrates that higher S1, lower CAR and higher cortisol AUC measures were observed at measurement occasions where participants in the aMCI group reported a higher amount of life event-related stress than on other occasions, but this association was absent in the control group.

### Cognitive decline in aMCI and control group

Table 3 shows a comparison of the rate of cognitive decline for both the primary outcome (FCSRT-IR) and secondary outcome (MOCA) in the aMCI and control group. There was no significant change in either cognitive measure in the control group over time. However, there was a significantly greater cognitive decline in both the FCSRT-IR and MOCA over time in the aMCI group compared with the control group.

**Table 3:** Cognitive decline in aMCI and control group for primary and secondary outcome.

	Outcome: FCSRT-IR			Outcome:MOCA		
	B	95% CI	P	B	95% CI	P
Intercept (control group)	47.51	45.43,49.60	<0.001	27.87	27.33,28.42	<0.001
Intercept (difference aMCI)	−9.39	−11.95,−6.82	<0.001	−5.10	−5.77,−4.42	<0.001
Time (years; control group)	0.02	−0.89,0.093	0.96	0.19	−0.24,0.61	0.39
Time (years; difference aMCI)	−2.73	−3.91,−1.56	<0.001	−1.24	−1.79,−0.69	<0.001

FCSRT-IR = Free and Cued Selective Reminding Test with immediate recall; MOCA = Montreal Cognitive Assessment.

Table 4 shows the relationship between the demographic variables and rate of cognitive decline as measured by the FCSRT-IR and MOCA. In aMCI subjects, females and carriers of the APOE e4 allele had significantly lower cognitive scores as measured by the FCSRT-IR at baseline. Carriers of the APOE e4 allele also had significantly higher rates of cognitive decline as measured by change in the FCSRT-IR. In aMCI subjects, females but not APOE e4 allele carriers had significantly lower MOCA scores at baseline but there was a marginally significant (p = 0.054) relationship between carriers of the APOE e4 allele and higher rates of cognitive decline as measured by change in the MOCA.

**Table 4:** Estimated fixed effects for cognitive decline in FCSRT-IR and MOCA in the aMCI group and influence of age, gender and APOE e4-status.

	Model 1			Model 2		
	B	95% CI	P	B	95% CI	P
**FCSRT-IR**						
Intercept	38.13	36.30,39.96	<0.001	42.86	40.09,45.64	<0.001
Time (years)	−2.73	−3.70,−1.77	<0.001	−1.05	−2.50,0.40	0.15
Age at baseline				−0.14	−0.36,0.09	0.23
Gender (female)				−5.94	−9.50,−2.38	0.001
Gender x time				−1.25	−3.25,0.76	0.22
APOE-e4 (present)				−4.58	−8.08,−1.08	0.010
APOE-e4 x time				−2.65	−4.57,−0.74	0.006
**MOCA**						
Intercept	22.79	22.35,23.23	<0.001	23.80	23.13,24.47	<0.001
Time (years)	−1.14	−1.56,−0.72	<0.001	−0.55	−1.18,0.08	0.086
Age at baseline				−0.08	−0.13,−0.03	0.003
Gender (female)				−1.76	−2.62,−0.90	<0.001
Gender x time				−0.38	−1.24,0.49	0.39
APOE-e4 (present)				−0.34	−1.18,0.51	0.44
APOE-e4 x time				−0.81	−1.64,0.02	0.054

MOCA = Montreal Cognitive Assessment; FCSRT-IR = Free and Cued Selective Reminding Test with immediate recall; ApoE ε4 = Apolipoprotein E ε4 allele carrier.

### Influence of stress on cognitive decline

Table 5 shows the relationship between stress variables and rates of cognitive decline in aMCI participants as measured by the FCSRT-IR. Within subject multivariate analysis shows no significant relationships between PSS, RLCQ or the salivary cortisol measures S1 and CAR with baseline FCSRT-IR scores. However, there was a significant negative relationship between baseline cortisol AUC levels and baseline FCSRT-IR such that higher cortisol AUC levels were associated with lower scores on the FCSRT-IR. However, over time, this negative relationship was reversed and high levels of cortisol AUC were associated with higher scores on the FCSRT-IR, i.e. subjects with higher cortisol AUC levels had a slower rate of cognitive decline as measured by the FCSRT-IR. Between subject multivariate analysis shows that higher average (measured over all four visits) PSS scores for each individual were associated with higher average (measured over all four visits) FCSRT-IR scores. Females had lower FCSRT scores at baseline. Subjects carrying the APOE e4 allele had lower average FCSRT scores and, over time, subjects carrying the APOE e4 allele had lower FCSRT scores compared to non carriers i.e. APOE e4 carriers had a greater rate of cognitive decline as measured by the FCSRT-IR.

**Table 5:** Cognitive decline as measured by the FCSRT-IR and influence of stress variables.

	Univariate			Multivariate		
	B	95% CI	P	B	95% CI	P
Intercept	Model dependent			44.54	29.67,59.42	<0.001
Time (years)	Model dependent			−2.42	−10.79,5.95	0.57
**Within-subjects**						
PSS	0.012	−0.18,0.21	0.90	−0.19	−0.43,0.04	0.11
PSS x time	−0.09	−0.34,0.16	0.48	0.11	−0.18,0.40	0.46
RLCQ	−0.003	−0.012,0.006	0.52	−0.006	−0.018,0.005	0.30
RLCQ x time	−0.008	−0.019,0.005	0.22	−0.007	−0.020,0.008	0.36
S1	−0.29	−2.25,1.66	0.77	1.34	−1.44,4.12	0.34
S1 x time	0.88	−1.43,3.18	0.75	−1.30	−4.62,2.02	0.44
CAR	0.12	−2.06,2.31	0.11	2.37	−0.43,5.17	0.097
CAR x time	−0.47	−2.76,1.82	0.69	−2.20	−5.11,0.71	0.14
cortisol AUC	−0.02	−0.039,0.005	0.13	−0.03	−0.06,−0.01	0.009
cortisol AUC x time	0.03	0.002,0.064	0.035	0.05	0.02,0.08	0.004
**Between-subjects**						
PSS	0.20	−0.12,0.52	0.22	0.54	0.21,0.86	0.001
PSS x time	−0.05	−0.23,0.13	0.57	−0.07	−0.25,0.11	0.45
RLCQ	0.003	−0.018,0.024	0.76	−0.007	−0.029,0.015	0.52
RLCQ x time	−0.013	−0.025,−0.000	0.045	−0.001	−0.014,0.012	0.87
S1	−0.05	−3.24,3.15	0.98	−2.50	−7.62,2.61	0.34
S1 x time	0.30	−1.61,2.21	0.76	1.56	−1.49,4.61	0.32
CAR	0.49	−3.31,4.28	0.80	−0.68	−6.49,5.12	0.82
CAR x time	0.98	−1.22,3.18	0.38	2.55	−0.93,6.03	0.15
cortisol AUC	−0.004	−0.033,0.026	0.82	−0.002	−0.036,0.031	0.90
cortisol AUC x time	−0.009	−0.031,0.012	0.40	−0.012	−0.040,0.016	0.39
Age				−0.18	−0.44,0.08	0.17
Gender (female)				−7.99	−11.82,−4.16	<0.001
Gender x time				−1.60	−3.82,0.62	0.16
APOE-e4 (present)				−5.18	−9.05,−1.32	0.009
APOE-e4 x time				−2.17	−4.26,−0.08	0.042

RLCQ = Recent Life Changes Questionnaire; PSS = Perceived Stress Scale; S1 = Awakening cortisol sample; CAR = Cortisol awakening response; cortisol AUC = area under daytime cortisol curve; ApoE ε4 = Apolipoprotein E ε4 allele carrier.

Table 6 examining the MOCA shows similar findings except that the significant relationship between salivary cortisol measures is with the CAR and not the cortisol AUC. Thus, there was a significant negative relationship between baseline CAR levels and baseline MOCA scores within subjects, such that higher CAR levels at baseline were associated with lower scores on the MOCA. However, over time, this negative relationship was reversed and high CAR associated with higher scores on the MOCA, i.e. subjects with high CAR had a slower rate of cognitive decline as measured by the MOCA. In addition, no significant relationship was found between APOE e4 carriers and baseline MOCA scores or changes in MOCA scores over time.

**Table 6:** Cognitive decline as measured by the MOCA and influence of stress variables.

	Univariate			Multivariate		
	B	95% CI	P	B	95% CI	P
Intercept	Model dependent			22.56	18.85,26.27	<0.001
Time (years)	Model dependent			0.016	−4.14,4.17	0.99
Within-subjects						
PSS	−0.03	−0.10,0.05	0.47	−0.027	−0.113,0.059	0.54
PSS x time	−0.005	−0.099,0.089	0.91	0.033	−0.077,0.143	0.56
RLCQ	−0.002	−0.005,0.002	0.38	0.0003	−0.004,0.005	0.88
RLCQ x time	0.002	−0.003,0.006	0.41	0.0018	−0.003,0.007	0.50
S1	−0.02	−0.71,0.68	0.97	−0.46	−1.51,0.58	0.39
S1 x time	−0.26	−1.08,0.56	0.53	0.43	−0.84,1.71	0.51
CAR	−0.59	−1.36,0.17	0.13	−1.24	−2.30,−0.18	0.022
CAR x time	0.82	0.02,1.62	0.044	1.25	0.11,2.32	0.032
cortisol AUC	0.001	−0.007,0.009	0.81	0.002	−0.007,0.011	0.65
cortisol AUC x time	−0.003	−0.014,0.008	0.57	−0.005	−0.017,0.007	0.43
Between-subjects						
PSS	0.019	−0.057,0.096	0.62	0.068	−0.014,0.150	0.10
PSS x time	−0.04	−0.11,0.04	0.35	0.017	−0.075,0.109	0.71
RLCQ	−0.002	−0.007,0.003	0.35	−0.004	−0.009,0.002	0.20
RLCQ x time	−0.003	−0.009,0.002	0.24	−0.0004	−0.007,0.006	0.90
S1	0.47	−0.29,1.22	0.23	−0.04	−1.327,1.247	0.96
S1 x time	−0.06	−0.88,0.76	0.89	0.05	−1.46,1.56	0.95
CAR	−0.18	−1.08,0.72	0.70	0.52	−0.93,1.98	0.48
CAR x time	0.11	−0.83,1.06	0.81	0.39	−1.32,2.10	0.65
cortisol AUC	0.004	−0.004,0.011	0.34	0.006	−0.002,0.015	0.15
cortisol AUC x time	−0.004	−0.014,0.005	0.36	−0.008	−0.021,0.006	0.28
Age				−0.05	−0.12,0.01	0.11
Gender (female)				−2.04	−3.00,−1.07	<0.001
Gender x time				−0.47	−1.58,0.65	0.41
APOE-e4 (present)				−0.21	−1.17,0.76	0.68
APOE-e4 x time				−0.49	−1.53,0.55	0.36

RLCQ = Recent Life Changes Questionnaire; PSS = Perceived Stress Scale; S1 = Awakening cortisol sample; CAR = Cortisol awakening response; cortisol AUC = area under daytime cortisol curve; ApoE ε4 = Apolipoprotein E ε4 allele carrier.

### Relationship between stress measures and conversion to dementia

**Figure 2:**
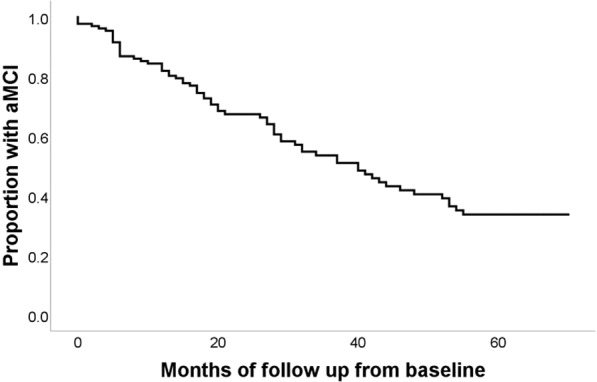
Kaplan-Meier survival plot for aMCI subjects developing Dementia from baseline.

Subjects with aMCI were followed up for a mean period of 28.0 (s.d. 20.7) months. At the end of the 5.5 year follow up period; 66 (50%) participants of the aMCI population had developed dementia, 7 (5%) had died and 42 (32%) were lost to follow up. The median time of conversion to dementia was 40.0 months. None had converted to normal cognition. Figure 2 shows a Kaplan-Meier survival plot in aMCI subjects developing Dementia.

Table 7 shows a Cox hazard survival analysis of aMCI subjects developing Dementia using demographic, cognitive scores and stress variables in both univariate and multivariate analysis. Univariate analysis shows increased age to be a risk factor for the development of Dementia, whilst a high FCSRT-IR; MOCA and S1 cortisol measures at baseline were protective. Correcting for potential confounding between these variables multivariate analysis showed that only a higher FCSRT-IR and a higher MOCA score remained significantly independently related to the development of dementia, both of which appeared protective.

**Table 7:** Survival analysis: time to conversion to Dementia in patients with amnestic mild cognitive impairment at baseline.

Variable name	Univariate			Multivariate		
	Hazard Ratio	95% CI	P	Hazard Ratio	95% CI	P
Age (year)	1.06	1.02,1.10	0.002	1.039	0.99,1.09	0.12
Gender (female)	0.63	0.39,1.01	0.06	1.643	0.81,3.35	0.17
ApoE E4 (present)	0.94	0.56,1.56	0.80	0.539	0.23,1.24	0.15
FCSRT-IR	0.95	0.93,0.97	<0.0001	0.957	0.92,0.99	0.02
MOCA	0.81	0.73,0.89	<0.0001	0.833	0.71,0.98	0.03
RLCQ	0.99	0.99,1.00	0.31	0.999	0.99,1.00	0.40
PSS	0.98	0.95,1.02	0.31	1.027	0.98,1.08	0.29
S1	0.57	0.37,0.87	0.01	0.539	0.26,1.14	0.10
CAR	1.50	0.88,2.44	0.14	0.792	0.35,1.77	0.57
cortisol AUC	1.00	0.99,1.00	0.95	1.004	1.00,1.01	0.06

MOCA = Montreal Cognitive Assessment; FCSRT-IR = Free and Cued Selective Reminding Test with immediate recall; RLCQ = Recent Life Changes Questionnaire; PSS = Perceived Stress Scale; S1 = Awakening cortisol sample; CAR = Cortisol awakening response; cortisol AUC = area under daytime cortisol curve; ApoE ε4 = Apolipoprotein E ε4 allele carrier.

## Discussion

In this study, we examined the relationship of three stress measures, an objective life event measure (RLCQ), a measure of distress (PSS) and three aspects of salivary cortisol measurements and examined their relationship with baseline cognition, cognitive decline and conversion to dementia in an aMCI population. We hypothesised that our primary measure of stress, the RLCQ and secondary measures (PSS and salivary cortisol measures) would be associated with a faster rate of cognitive decline and an increased conversion rate to dementia.

Half of the participants with aMCI converted to dementia over the 5.5 year study. This is a relatively high conversion rate but is comparable to findings from a metanalysis of 13 studies showing a range of between 29 to 50% conversion to dementia in a specialist setting^31^.

As shown in other studies^32,33^, measures of distress (PSS) were markedly increased in the aMCI group compared with the control cohort. These findings emphasise the high degree of psychiatric co-morbidity and the potential need for clinical interventions to alleviate distress in this population. However, contrary to our expectations we found no relationship between PSS or RLCQ with increased rate of cognitive decline or conversion to dementia up to 5.5 years later. However, we did find a relationship between cortisol measures and cognitive outcomes. Thus, high levels of cortisol (measured as cortisol AUC or CAR) at baseline were related to poorer cognitive performance at baseline. Thus, subjects with low FCSRT-IR cognitive scores at baseline had higher cortisol AUC measures at baseline and those with low MOCA cognitive scores at baseline had higher CAR measures. Notably, although not significant we also found a trend (p = 0.05) relationship showing an increased cortisol AUC Levels in the aMCI group compared with the control group after correction for age and gender. These findings are thus consistent with the hypothesis that high levels of cortisol may have had a damaging effect on the hippocampus and are consistent with studies suggesting a potential role of cortisol in precipitating the early development of aMCI^34^.

However, when we followed individuals over time we found that cortisol measurements dropped in direct relationship to the diminishing level of cognitive performance at each successive visit i.e. as the FCSRT-IR declined so did the cortisol AUC. Likewise, over time changes in the MOCA parallel changes in the CAR i.e. as the MOCA declined so did the CAR. This is an unexpected finding since hippocampal atrophy might be expected to increase cortisol levels because the hippocampus exerts an inhibitory effect on the HPA axis^35^. However, this apparently contradictory finding was also found in the only other longitudinal study of aMCI subjects^21^ as so our data supports this finding. One interpretation of this finding is that in aMCI subjects the existing hippocampal damage changes the impact of cortisol on hippocampal function and memory^21^. However, another interpretation is that the hippocampus, as well as being susceptible to damage by high cortisol levels, may, regulate production of cortisol levels to stressful situations. Thus, a blunted cortisol response to psychological stress has been shown to be present when there is hippocampal damage,^36–40^ and so the reduced cortisol response to psychological stress may be due to the failing hippocampal reactivity to stressful events in the aMCI group. In our study, the CAR, is the only cortisol measure that at least partly indicate cortisol reactivity. Our finding that objective life stress as measured by the RLCQ was negatively associated with the CAR in the aMCI group but not the control group is in line with this interpretation. Although there was a trend relationship (p = 0.06) between increased cortisol AUC cortisol measures at baseline and increased conversion to dementia we did not find any other indicators that cortisol measures were associated with increased conversion to dementia. A recent study has suggested that a single measure of plasma cortisol, in conjunction with other plasma and CSF measures, may be a biomarker for conversion to Alzheimer’s Disease in a MCI population^41^. Our results suggest that the impact of cortisol measures on cognitive decline in aMCI subjects may become less as the disease progresses and so this relationship may alter as the disease progresses towards Alzheimer’s disease.

A range of cortisol measures in this study showed a significantly positive within-subject association with RLCQ scores in the aMCI group and a significantly positive between-subject association with the PSS score. However, these between and within-subject effects appear to be largely confounded by age, and the increasing blunted response between the psychometric measures of stress and cortisol response as the hippocampus atrophies may, in part, explain why we found no relationship between the psychometric measures of stress and cognitive decline in the aMCI group. As shown elsewhere we confirm that the allele ApoE ε4 is associated with both the presence of aMCI and increased rate of cognitive decline in this group^42,43^.

Several limitations to this study should be noted. Contemporary definitions of Mild Cognitive Impairment identify a number of clinical subtypes including impairments in non-memory cognitive domains^44^ and the restricted use of the aMCI definition^45^ in this study does not address this heterogeneity. However, we wanted to examine a group that was a potential group for targeted intervention and so a more restricted aMCI definition was utilised. Life time medical or psychological co-morbidities (including disturbed sleep pattern) were not available for analysis and so could not be assessed as possible confounding variables. The use of stress questionnaires carry limitations including a restricted number and type of stressful events; retrospective forgetting of events and reporting bias that may influence sensitivity of such scales. Although salivary cortisol is a commonly used measure in stress research^46^ its reliability as a measure has often been questioned due to a number of variables shown able to influence daily levels^47,48^. In addition, it should be noted that our models for predicting scores on measures of cognitive function included a rather large number of predictors and therefore are susceptible to the risk of overfitting. Although the results were quite stable in univariate and multivariate models, and the cortisol findings were in line with an earlier study as discussed above, our findings need to be supported by future replication studies. Finally, diagnoses in this study were symptom based without the use of amyloid diagnostic scans; other neurological scans or CSF markers that would have enabled a more detailed assessment of diagnostic subtypes and so the characterisation of these subjects was limited.

## Methods

### Standard protocol approvals, registrations and patient consents

Approval for the research was granted by a multi-centre research ethics committee (NRES Committee South Central – Portsmouth 12/SC/0115) and was registered with the National Institute for Health Research (96678). All research methods were performed in accordance with the relevant guidelines/regulations including the Declaration of Helsinki and the principles of Good Clinical Practice. Informed consent was obtained from all participants.

### Study design and participants

This was an 18 month longitudinal observational multi-centre cohort assessing the relationship between stress and rates of cognitive decline in both aMCI and cognitively intact participants. aMCI participants were then followed annually for a further 4 years to assess conversion to dementia. Site initiation meetings and training were delivered at all sites to ensure compliance with the standardised protocol methodology.

The following psychological measures of stress were assessed six monthly over the first 18 months of the study. The RLCQ^27^ was our primary objective measure of stress. The RLCQ assesses stressful, neutral, and positive life events in five domains: health, home/family, financial, personal/social and work with each life event item given a weighting. High scores indicate high adverse life events. The PSS was a measure of global perceived stress (distress). Participants are required to subjectively rate how often they have experienced certain feelings or thoughts of stress over the previous month on a 0 to 4 Likert scale. High scores indicate high levels of perceived stress^28^. Salivary cortisol was used as a biological measure of stress. Three aspects of cortisol, the first salivary cortisol measure taken as soon as possible after wakening (s1), the cortisol awakening response (CAR) measuring the post-awakening surge in cortisol occurring within 30 minutes after wakening and the area under the daytime cortisol curve (cortisol AUC), an estimate of the average daily cortisol exposure, were measured. Our primary measure of cognition was the Free and Cued Selective Reminding Test (FCSRT-IR) which measures impairment of episodic memory under conditions that control for attention and cognitive processing^29^. The Montreal Cognitive Assessment (MocA)^30^ was used as a secondary measure for screening and to assess global cognitive function. At the end of the 18 month study aMCI participants were invited to take part in a four year extension to the study that established, on a yearly basis, if the diagnosis of aMCI remained or if the participant had either reverted to a diagnosis of normal cognition or had converted to dementia.

At screening, all eligible participants had to be aged 50 years or older; have adequate visual and auditory acuity to allow cognitive testing; be capable of giving informed consent. Participants with aMCI had to have received a prior provisional diagnosis of mild cognitive impairment from an NHS clinician and have a study partner spending at least 10 hours per week with the participant. Final diagnosis of aMCI was based on fulfilment of Petersen criteria for aMCI^45^ as assessed by the consensus opinion of two research doctors (CH; RS) and utilised a range of cognitive measures in addition to the MOCA including the FCSRT-IR; an assessment of verbal fluency^49^; a digit symbol substitution test^50^ and a trail making test^51^. Functional activities were assessed using the Medical Outcomes Study-Social Support Survey^52^. Eligible control participants had to: have no objective memory problems with a MoCA score at baseline of greater than 24 points. All participants taking cognitive enhancers, e.g. cholinesterase inhibitors or memantine or major modifiers of the immune system e.g. corticosteroids or TNFα inhibitors, at baseline or throughout the first 18 months of the study were excluded. All participants were recruited from the South Coast of England, UK. 133 aMCI and 68 control participants fulfilled the study entry criteria. 172 (86%) participants were recruited from Southampton (Memory Assessment and Research Centre; Moorgreen Hospital; Southern Health Foundation Trust), 20 (10%) from Portsmouth (St Mary’s Community Health Campus, Solent health care trust) and 9 (4%) from Dorset (Sherbourne study centre, Dorset Healthcare University NHS Foundation Trust) between April 2013 and February 2014 between April 2013 and February 2014.

### Procedures

Following consent all participants were screened for exclusion and inclusion criteria. Following inclusion, all participants (and study partners for participants with aMCI) were interviewed at baseline for information on recent (past 6 months) medical history. In addition, aMCI partner participants were interviewed regarding the occurrence of life events or symptoms in the aMCI participant over the previous six months using the RLCQ^27^. Participants with aMCI were then assessed using the FCSRT-IR, MoCA and the PSS. Following inclusion, control participants were interviewed with the RLCQ regarding events or symptoms in the previous 6 months and then assessed using the FCSRT-IR, MoCA and PSS. All questionnaires and assessments were repeated at 6, 12 and 18 months. The FCSRT-IR test has three versions. We used all three different versions, in the same order for each subject to reduce practice effects. Participants with aMCI who developed dementia defined using ICD-10 diagnostic criteria^53^ were excluded from further participation as required by the ethics committee and started on appropriate treatment.

At baseline blood samples were taken for DNA analysis (principally for ApoE ε4) from aMCI and control participants. ApoE genotypes were determined by Taqman genotyping of SNP rs7412 and KASP genotyping of SNP rs429358. Saliva samples collected by Salivette (Sarstedt, Nümbrecht, Germany) were taken at baseline, 6, 12 and 18 months at six time points (awakening; 30 mins after awakening; 11.00am, 3 pm, 6 pm and 9 pm) for cortisol analysis. Samples were refrigerated at the participants home before being transported in a cool box and stored at −80 °C. Saliva samples were sent in one batch to the Biochemical Laboratory at the Division of Theoretical and Clinical Psychobiology, University of Trier, Germany, where analysis for free cortisol content (nmol/L) was by time-resolved immunoassay with fluorescent detection^54^. The detection limit for the assay was 0.173 nmol/L. Each sample was measured in duplicate, with an intra-assay coefficient of variance between 4.0% and 6.7%, and inter-assay coefficient of variance between 6.4% and 8.9%. Cortisol awakening response (CAR) was assessed as the difference between the immediate awakening sample and the sample taken 30 minutes later^55^. Daily cortisol level was assessed by the area under the curve of all cortisol measures after excluding the 30 minutes after awakening sample to avoid confounding with the CAR.

Clinical diagnoses were reviewed annually by research doctors (CH; RS) following the end of the initial 18 month study. Medical information was obtained by a review of all hospital; clinical research and general practitioner notes.

### Statistical analysis

The primary outcome measure was the relationship between the RLCQ and rate of cognitive decline as measured by change in the FCSRT-IR during an 18 month follow up period in aMCI compared to control participants. All assessment dates were equally spaced at 6 monthly intervals. Power calculations were based on our primary outcome measure. Previous research^21^ has shown that approximately 50% of participants with aMCI will experience negative life events over an 18 month follow up period. One hundred participants with aMCI gave 80% power to detect a significant (α = 0.05) decrease of 0.5 s.d. points in the FCSRT-IR in the aMCI group experiencing negative life events compared with the group without negative life events. The rationale for powering the study to examine a change of 0.5 s.d. of the change in the FCSRT-IR was based on a systematic review of studies^56^, supported in dementia research^57^, that showed that meaningful clinical important differences for health related life measures show a convergence upon a value of 0.5 s.d.^58^. We assumed no significant longitudinal changes in cognition on the FCSRT-IR in the control group regardless of life events. Allowing for a 25% drop out rate meant we required 134 participants with aMCI. The control group size of 68 was based on 0.8 power to detect mean differences (s.d. 0.5 points) at baseline [α = 0.01 to adjust the nominal significance (p < 0.05) for the five main comparisons] in the psychological stress parameters (RLCQ, PSS) and physiological stress measures (s1, CAR and daily salivary cortisol) in the aMCI group compared to the control group. Study demographic characteristics, efficacy measure outcomes and serum and salivary proteins were assessed for normality using Q-Q plots. Non-normal data was log_10_ transformed.

Associations between repeated measures were analysed using maximum likelihood fitted mixed-effects regression models with a random intercept, also including a random effect for time in those models that included time as a predictor. Cognitive decline models used MOCA and FCSRT-IR scores as outcome variables and included time as well as the interaction of time with other predictor variables to model linear trends across the observation period. These models were limited to the aMCI group, as there was virtually no cognitive decline observed in the control group. Within- and between-subject effects were separated by centering observations within subjects around the person specific mean. Both, within- and between-subject (i.e. person-specific mean) variables were entered in each model. Time was measured in years at the exact date of assessment and centered at the baseline visit (i.e. time at baseline = 0). Age was centered at the sample’s mean age at baseline (74.5 years). We do not show variances of random effects and residuals in the results tables as our focus was on average associations in the sample (i.e. fixed effects).

All statistical analyses were performed in Microsoft Excel 2003 (Microsoft Corporation, Redmond, WA), Stata version 15.1 (StataCorp, College Station, TX), and SPSS version 24 (SPSS software, IBM Corporation, Armonk, NY).

The datasets generated during and/or analysed during the current study are available from the corresponding author on reasonable request.

## Supplementary information

Supplemental file.
