# Supplementary material for: Psychological stress, cognitive decline and the development of dementia in amnestic mild cognitive impairment
Source: Sci Rep. 2020 Feb 27;10:3618. doi: 10.1038/s41598-020-60607-0 (PMC7046646; doi:10.1038/s41598-020-60607-0)
Supplement: Supplementary file 1 — Supplemental file. [file 41598_2020_60607_MOESM1_ESM.pdf]

Psychological stress, cognitive decline and the development of dementia in amnestic mild cognitive impairment

Rebecca Sussams[1, 2], Wolff Schlotz[3], Zoe Clough[1,2], Jay Amin[1, 2], Sharon Simpson[4], Amelia Abbott[1, 2], Rebecca Beardmore[1, 2], Richard Sharples[2], Rachel Raybould[5], Keeley Brookes[6], Kevin Morgan[6], David Culliford[7], Clive Holmes[1, 2*]

Supplemental Table 1. Between- and within-subject effects of stress and covariates on S1 cortisol measures (immediately after awakening, Box-Cox transformed – original in units of nmol/L). Group interactions represent differences between comparison and aMCI group.

	Model 1								Model 2						
	Univariate				Multivariate				Univariate				Multivariate		
Variable	B	95% CI	P		B	95% CI	P		B	95% CI	P		B	95% CI	P
Within-subjects															
PSS	0.0001	-0.01,0.01	.99		-0.003	-0.015,0.009	.61		-0.007	-0.026,0.011	.44		-0.005	-0.025,0.015	.64
RLCQ	0.0004	-0.0001,0.001	.13		0.001	-0.0001,0.001	.12		-0.0004	-0.001,0.0004	.37		-0.0003	-0.001,0.001	.52
Group x PSS									0.012	-0.012,0.037	.31		0.007	-0.019,0.032	.61
Group x RLCQ									0.002	0.0004,0.003	.007		0.002	0.000,0.003	.017
Between-subjects															
PSS	0.016	0.003,0.028	.013		0.018	0.005,0.031	.007		0.002	-0.017,0.020	.87		-0.003	-0.023,0.018	.81
RLCQ	0.0002	-0.001,0.001	.62		-0.0002	-0.001,0.001	.61		0.0002	-0.001,0.002	.76		0.001	-0.001,0.002	.71
Group x PSS									0.022	-0.003,0.047	.084		0.025	-0.003,0.051	.077
Group x RLCQ									0.0002	-0.002,0.002	.84		-0.001	-0.003,0.001	.49
Age (years)	-0.003	-0.01,0.01	.47		-0.006	-0.014,0.003	.17		0.009	-0.003,0.022	.16		0.010	-0.002,0.023	.11
Gender (Female)	-0.15	-0.30,0.001	.052		-0.179	-0.329,-0.028	.020		-0.148	-0.409,0.114	.27		-0.144	-0.284,0.097	.24
Group x age									-0.034	-0.052,-0.017	< .001		-0.035	-0.053,-0.017	< .001
Group x gender									0.044	-0.286,0.374	.80		-0.001	-0.309,0.308	.99

Supplemental Table 2. Between- and within-subject effects of stress and covariates on CAR measures (increase after awakening, Box-Cox transformed – original in units of nmol/L). Group interactions represent differences between comparison and aMCI group.

	Model 1						Model 2					
	Univariate			Multivariate			Univariate			Multivariate		
Variable	B	95% CI	P	B	95% CI	P	B	95% CI	P	B	95% CI	P
Within-subjects												
PSS	0.001	-0.007,0.018	.36	0.008	-0.005,0.020	.21	0.009	-0.010,0.028	.34	0.005	-0.015,0.026	.61
RLCQ	-0.0001	-0.001,0.0004	.67	-0.0002	-0.001,0.0004	.57	0.001	-0.0003,0.001	.18	0.0005	-0.0004,0.001	.27
Group x PSS							-0.006	-0.031,0.018	.61	0.001	-0.025,0.027	.96
Group x RLCQ							-0.001	-0.002,-0.0002	.023	-0.001	-0.002,-0.000	.045
Between-subjects												
PSS	-0.015	-0.026,-0.005	.004	-0.017	-0.027,-0.006	.002	-0.011	-0.026,0.005	.18	-0.010	-0.028,0.008	.27
RLCQ	-0.001	-0.001,0.0001	.099	-0.0002	-0.001,0.001	.68	-0.001	-0.002,0.001	.38	-0.0002	-0.001,0.001	.77
Group x PSS							-0.006	-0.027,0.015	.58	-0.006	-0.028,0.017	.63
Group x RLCQ							-0.0004	-0.002,0.001	.58	-0.0003	-0.002,0.001	.69
Age (years)	0.002	-0.005,0.009	.61	0.004	-0.003,0.011	.24	-0.002	-0.013,0.009	.68	-0.002	-0.013,0.008	.68
Gender (Female)	0.172	0.045,0.299	.008	0.185	0.061,0.309	.004	0.239	0.021,0.457	.031	0.248	0.046,0.450	.016
Group x age							0.017	0.001,0.032	.033	0.017	0.002,0.032	.031
Group x gender							-0.142	-0.418,0.134	.31	-0.150	-0.412,0.112	.26

Supplemental Table 3. Between- and within-subject effects of stress and covariates on AUC measures (diurnal cortisol area under the curve, Box-Cox transformed – original in units of nmol/L). Group interactions represent differences between comparison and aMCI group.

	Model 1						Model 2					
	Univariate			Multivariate			Univariate			Multivariate		
Variable	B	95% CI	P	B	95% CI	P	B	95% CI	P	B	95% CI	P
Within-subjects												
PSS	-0.354	-1.61,0.91	.58	-0.587	-1.89,0.72	.38	-0.27	-2.20,1.66	.78	0.38	-1.73,2.49	.73
RLCQ	0.035	-0.025,0.095	.25	0.042	-0.019,0.103	.18	-0.07	-0.15,0.02	.12	-0.08	-0.17,0.02	.11
Group x PSS							-0.15	-2.71,2.40	.91	-0.96	-3.66,1.73	.49
Group x RLCQ							0.20	0.08,0.32	.001	0.21	0.09,0.34	.001
Between-subjects												
PSS	1.40	0.19,2.60	.023	1.40	0.16,2.64	.026	0.31	-1.49,2.10	.74	0.25	-1.77,2.28	.81
RLCQ	-0.005	-0.09,0.08	.91	-0.02	-0.11,0.07	.62	-0.009	-0.131,0.114	.89	0.006	-0.135,0.147	.93
Group x PSS							1.53	-0.90,3.96	.22	1.30	-1.33,3.92	.33
Group x RLCQ							0.026	-0.143,0.194	.77	-0.006	-0.186,0.174	.95
Age (years)	0.62	-0.21,1.45	.14	0.40	-0.43,1.23	.35	1.18	-0.08,2.43	.066	1.14	-0.12,2.39	.075
Gender (Female)	-8.75	-23.67,6.17	.25	-9.56	-24.15,5.02	.20	-18.63	-43.78,6.53	.15	-17.55	-41.25,6.15	.15
Group x age							-2.06	-3.88,-0.23	.027	-1.77	-3.60,0.07	.059
Group x gender							25.08	-6.93,57.10	.13	18.01	-12.59,48.61	.25
